# Improved Low-Glucose Predictive Alerts Based on Sustained Hypoglycemia: Model Development and Validation Study

**DOI:** 10.2196/26909

**Published:** 2021-04-29

**Authors:** Darpit Dave, Madhav Erraguntla, Mark Lawley, Daniel DeSalvo, Balakrishna Haridas, Siripoom McKay, Chester Koh

**Affiliations:** 1 Department of Industrial and Systems Engineering Texas A&M University College Station, TX United States; 2 Department of Pediatrics Baylor College of Medicine / Texas Children's Hospital Houston, TX United States; 3 Department of Biomedical Engineering Texas A&M University College Station, TX United States; 4 Division of Pediatric Urology Texas Children's Hospital Houston, TX United States; 5 Scott Department of Urology Baylor College of Medicine Houston, TX United States

**Keywords:** machine learning, quantile regression forests, sustained hypoglycemia, continuous glucose monitoring, false alert rate, model generalizability, diabetes, glucose monitoring, hypoglycemia

## Abstract

**Background:**

Predictive alerts for impending hypoglycemic events enable persons with type 1 diabetes to take preventive actions and avoid serious consequences.

**Objective:**

This study aimed to develop a prediction model for hypoglycemic events with a low false alert rate, high sensitivity and specificity, and good generalizability to new patients and time periods.

**Methods:**

Performance improvement by focusing on sustained hypoglycemic events, defined as glucose values less than 70 mg/dL for at least 15 minutes, was explored. Two different modeling approaches were considered: (1) a classification-based method to directly predict sustained hypoglycemic events, and (2) a regression-based prediction of glucose at multiple time points in the prediction horizon and subsequent inference of sustained hypoglycemia. To address the generalizability and robustness of the model, two different validation mechanisms were considered: (1) patient-based validation (model performance was evaluated on new patients), and (2) time-based validation (model performance was evaluated on new time periods).

**Results:**

This study utilized data from 110 patients over 30-90 days comprising 1.6 million continuous glucose monitoring values under normal living conditions. The model accurately predicted sustained events with >97% sensitivity and specificity for both 30- and 60-minute prediction horizons. The false alert rate was kept to <25%. The results were consistent across patient- and time-based validation strategies.

**Conclusions:**

Providing alerts focused on sustained events instead of all hypoglycemic events reduces the false alert rate and improves sensitivity and specificity. It also results in models that have better generalizability to new patients and time periods.

## Introduction

Glucose measurements are critical for effective diabetes management. Real-time continuous glucose monitoring (CGM) devices allow for frequent, automated glucose readings from interstitial fluid in the subcutaneous tissue space. CGM has been shown to improve glycemic control and reduce glycemic excursions—decreasing both hypoglycemia and hyperglycemia [1]. An important feature of CGM devices is their ability to provide real-time auditory alerts for trending glucose excursions above or below customized threshold levels. The CGM data can also be used to develop models to predict future hypoglycemia [1,2].

Since the first attempt at predicting future glucose values based on CGM data in 1999 [3], numerous studies have been performed to provide alerts for hypoglycemia based on predictive models. Some of the initial works relied on classical time-series based forecasting methods such as autoregressive integrated moving average (ARIMA)–ARIMAX [4-9] and state-space models [10-19]. Later, different machine learning methods were explored to predict future glucose values [20-25]. More recently, deep learning techniques have been proposed toward solving the problem [26-33]. Some works have also explored incorporating contextual data such as meal information, insulin levels, heart rate, and physical activity as features in the predictive model [34-40]. However, currently integrated information on the data sources is not readily available for real-time predictions.

Predictive hypoglycemia alerts have the potential to be extremely helpful in reducing hypoglycemia risk; however, false alerts have been a major hindrance to the acceptance of predictive hypoglycemia alerts among users [41-43]. In our earlier work, we developed a machine learning–based hypoglycemia predictive model with a sensitivity and specificity of >95%, comparable with the best predictive models in the literature [44]. Typically, the number of hypoglycemic events is very small compared with that of nonhypoglycemic events. For example, only 2.13% (35,075/1,644,875) of readings in the data set used in this study were in the hypoglycemic range (ie, <70 mg/dL). The high-class imbalance resulted in a false alert rate (FAR) of around 85% (40,502/47,683), even with an impressive specificity of 95%. Improvement in specificity in such highly imbalanced class cases will reduce the FAR and therefore improve user experience and trust in the alerts, facilitating persuasive adoption of alerts.

Previous studies have found that hypoglycemia prediction model performance is reduced when applied to new patients and different time periods [45]. Improvement in model generalizability to new patients and time periods will facilitate ease of deployment and retention of performance postdeployment.

Thus, despite the many advances made in terms of hypoglycemia prediction models, the shortcoming of a high FAR makes the alerts ill-suited for real-world application [42,46,47]. These results indicate a need for the development of approaches to reduce the FAR, maintain high sensitivity, and improve the generalizability of the prediction model.

## Methods

### Data Description

The CGM data sets were obtained from 110 pediatric patients with type 1 diabetes over 30 to 90 days. The data comprised over 1.6 million CGM values under normal living conditions. Dexcom G6 CGM devices were used to collect the CGM readings. The cohort-level profile of patients in this study can be found in Table 1. Summary statistics of a patient hypoglycemia profile and a patient pump profile are presented in Multimedia Appendices 1 and 2, respectively. Of note, data were obtained from a mix of patients using multiple daily injections, sensor-augmented pump therapy without automated basal rate modulation, and sensor-augmented pump therapy with a predictive low-glucose suspend feature (ie, t:slim X2 insulin pump with Basal-IQ technology; Tandem Diabetes Care, Inc [48]). The t:slim X2 insulin pump uses a simple linear regression algorithm to predict glucose levels 30 minutes ahead and suspend basal insulin in the pump if glucose values are predicted to drop below 80 mg/dL in the next 30 minutes or if a CGM value falls below 70 mg/dL. Insulin delivery can remain suspended for a minimum of 5 minutes to a maximum of 2 hours and will then resume as soon as glucose values begin to rise.

**Table 1:** Demographic and diabetes profile of patients enrolled in the study.

Characteristic			Mean (SD)		Minimum		Maximum
**Baseline demographics**							
	Age (years)	12.67 (4.84)		1		21	
	Glycated hemoglobin A_1c_ (%)	7.70 (1.63)		5.00		12.50	
	Duration of diabetes (years)	4.93 (4.09)		0.25		19.18	
**Continuous glucose monitoring metrics**							
	Number of hypoglycemic values per day per patient	6.20 (5.98)		0.10		23.73	
	Percentage of hypoglycemic values below 70 mg/dL	2.13 (2.10)		0.50		12.20	

### Hypoglycemic Events

A glucose threshold of 70 mg/dL is used to identify the hypoglycemic range [49,50]. Of the 1.6 million CGM readings in the study data, approximately 35,000 values representing 6010 events were in the hypoglycemic range. The period of time between a first CGM reading below the threshold and the point where the CGM value rises to ≥70 mg/dL is considered a single “hypoglycemic event.”

Table 2 shows the frequency distribution of the duration of the hypoglycemic events. The hypoglycemic events in this analysis were further classified as transient (lasting less than 15 minutes; 77.24% [4642/6010] of the hypoglycemic events) or sustained (lasting 15 minutes or longer; 22.76% [1368/6010] of the events) based on the recommendations in previous studies [51,52]. Nonhypoglycemic events are CGM observations of ≥70 mg/dL. Detailed information regarding the distribution of hypoglycemic events and a breakdown of sustained events by day and night are presented in Multimedia Appendices 3-5.

**Table 2:** Frequency distribution of the duration of hypoglycemic events (n=6010).

Hypoglycemic events	Duration of consecutive CGM values falling below 70 mg/dL (minutes)									
	5	10	15	20	25	30	35	40	45	>45
Frequency, n (%)	572 (9.51)	796 (13.24)	885 (14.73)	842 (14.01)	676 (11.25)	562 (9.35)	391 (6.51)	283 (4.71)	209 (3.48)	794 (13.21)

### Evaluation Metrics

We define the following metrics for evaluating model performance: sensitivity, specificity, and FAR.

Sensitivity measures the proportion of true positives that are correctly identified. It is also known as the true-positive rate.

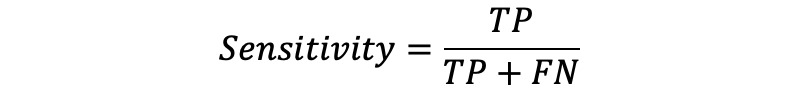



where TP are the true positives and FN are the false negatives.

Specificity measures the proportion of true negatives that are correctly identified. It is also known as the true-negative rate.

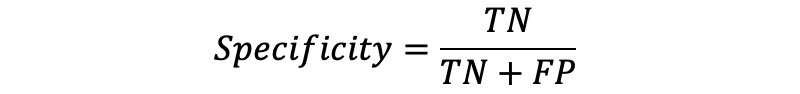



where TN are the true negatives and FP are the false positives.

FAR was defined based on the definition provided by Mosquera-Lopez et al [53] and measures the proportion of alerts that are not truly indicative of predicted hypoglycemic events.

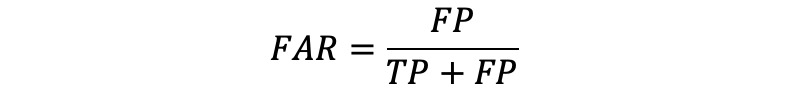



### Machine Learning Methodologies

#### Random Forest

Random forest (RF) is a nonparametric approach that builds on an ensemble prediction of a “forest” of regression trees grown via bootstrap sampling. Model predictions are obtained from the mean of the predictions of the individual trees. RF performs well when dealing with nonlinear relationships among variables and makes no assumptions about data distributions. Owing to these characteristics, utilizing RF-based machine learning modeling resulted in good performance in our previous work [44] compared with other machine learning methods for hypoglycemia prediction. In this study, an RF-based model was used to classify events as sustained hypoglycemic events (positive class) or transient and nonhypoglycemic events (negative class).

#### Quantile Regression Forest

For the multistep prediction approach, future CGM values were predicted using quantile regression forests (QRFs). The concept of quantile regression was introduced by Koenker and Hallock [54] and is advantageous when quantile functions are of interest. Quantile functions provide information about the spread of the response variable beyond the conditional mean by estimating the full conditional distribution. This is particularly useful for predicting values other than the mean (eg, median or 90th quantile). QRFs are a generalization of RFs and provides an accurate way of estimating the conditional quantiles [55]. Since it is more important to accurately predict CGM values near the hypoglycemic range rather than within the euglycemic or hyperglycemic range, QRFs were used to predict future CGM values using the regression approach.

QRFs were used as a multistep forecasting method to predict the glucose values for every 5-minute interval in the prediction horizon (PH). This resulted in 6 predictions for the 30-minute PH and 12 predictions for the 60-minute PH. Based on these predictions, a sustained hypoglycemic event was detected if 3 or more consecutive predicted CGM values were <70 mg/dL.

### Validation Mechanism

An appropriate validation mechanism is critical to assess the performance of a machine learning model [56,57]. This validation strategy helps to ascertain the generalizability of the model and ensures that the model performs well in real-world scenarios. This can be performed by sampling a subset of the data for model development and sampling a different sample of data for model validation [58]. Two validation strategies—patient-based and time-based—were used to evaluate model performance.

#### Patient-Based Approach

In this approach, the prediction model was developed on a subset of patients and validated on a different set of patients. Of the 110 patients, 70 patients (approximately 65% of the data) were randomly selected for training and the remaining 40 patients were used for performance evaluation. The final model performance reported is the mean of 5 replications of this procedure of 65%/35% split of training and validation data.

#### Time-Based Approach

In this approach, for each of the 110 patients, the first 70% of the data was used for model training and the last 30% of the data was used for validation. The average performance using validation data on all 110 patients was reported.

### Features Extracted for the Prediction

A rich combination of demographic, dynamic, snowball, interaction, and contextual features were extracted from the data. An optimal set of features for hypoglycemia prediction was identified in our previous work [44] and these features were used for the model development in this study (Multimedia Appendix 6). Records associated with missing data were eliminated from the analysis; that is, if a feature was dependent on a missing CGM value, that record—as well as all dependent time-lagged records—were eliminated from the analysis.

## Results

### Model Performance

Table 3 summarizes the performance of the model based on patient-based and time-based validation strategies. The total number of false alerts when considering transient and nonhypoglycemic events as false alerts and when considering nonhypoglycemic events as false alerts are provided along with sensitivity and specificity metrics.

In the patient-based validation approach, for both 30-minute and 60-minute PHs, the QRF method provided a significant advantage over the RF method with high sensitivity, high specificity, and low FAR. The patient-based validation approach indicated that the sustained hypoglycemic model developed using QRFs is generic and can be applied to new patients without performance degradation.

In a time-based validation setting, the RF method performed well for both 30-minute and 60-minute predictions with high sensitivity, high specificity, and low FAR, but the QRF method still outperformed it. The time-based validation methodology indicated that both models retain performance when applied to new time periods and in postdeployment.

**Table 3:** Comparison of model performance based on sensitivity, specificity, and false alerts with patient-based and time-based validation for 30-minute and 60-minute prediction horizons (PHs).

Metrics			Patient-based validation								Time-based validation						
			30-minute PH				60-minute PH				30-minute PH				60-minute PH		
			Method 1: RF^a^		Method 2: QRF^b^		Method 1: RF		Method 2: QRF		Method 1: RF		Method 2: QRF		Method 1: RF		Method 2: QRF
Sensitivity, % (SD)			39.11 (2.25)		99.09 (0.16)		49.27 (3.03)		97.61 (0.41)		96.17		98.94		95.34		97.91
Specificity, % (SD)			98.65 (0.09)		98.19 (0.10)		98.63 (0.12)		98.09 (0.11)		98.3		98.29		97.95		98.20
**False alerts, n (SD)**																	
	Considering transient and nonhypoglycemic events as false	6936 (356)		9339 (459)		7043 (317)		9672 (431)		6476		8211		7346		8465	
	Considering only nonhypoglycemic events as false	3907 (200)		5368 (162)		4109 (156)		5677 (201)		3324		4531		4334		4799	
False alert rate, % (SD)			26.32 (2.56)		26.50 (2.41)		26.44 (2.37)		26.36 (2.57)		22.79		23.86		26.41		23.79

^a^RF: random forest.

^b^QRF: quantile regression forest.

### Comparison of Sustained Versus All Hypoglycemic Events Prediction Models

Table 4 provides a comparison of model performance between sustained hypoglycemia and all-hypoglycemia prediction models. Even though the all-hypoglycemia model had high sensitivity, a specificity of 93% on a large number of nonhypoglycemic events resulted in an FAR of 85%. Focusing the alerts on sustained hypoglycemic events resulted in an increase in specificity to 98% and reduced the FAR to approximately 20% to 30%. Also, the performance of the all hypoglycemic events prediction model was adversely affected when evaluated on new patients and new time periods (drop in sensitivity of 5%). On the other hand, prediction models based on sustained hypoglycemic events retained their performance for new patients and new time periods, indicating better generalizability of the sustained hypoglycemic events model.

**Table 4:** Comparison of model performance based on sensitivity, specificity, and false alert rate with different characterizations of hypoglycemic events and different validation strategies (patient-based and time-based) for giving predictive alerts.

Model	30-minute prediction horizon				60-minute prediction horizon		
	Sensitivity (%)	Specificity (%)	False alert rate (%)	Sensitivity (%)		Specificity (%)	False alert rate (%)
All hypoglycemic events prediction (5-fold validation)	93.61	93.50	84.94	91.01		89.82	77.20
All hypoglycemic events prediction (new time periods)	87.10	92.66	85.16	73.87		87.29	79.81
All hypoglycemic events prediction (new patients)	87.60	92.47	75.20	73.79		87.06	71.50
Sustained hypoglycemic events prediction (QRF^a^—new patients)	99.08	97.79	30.00	98.13		97.58	30.19
Sustained hypoglycemic events prediction (QRF—new time periods)	98.54	98.57	22.36	97.72		98.49	22.44

^a^QRF: quantile regression forest.

A graphical comparison between the classifiers at different threshold values using receiver operating characteristic (ROC) curves can be found in Multimedia Appendix 7. The ROC plots show that the QRF model outperformed the RF models over the entire range of sensitivity and specificity levels. Table 5 shows the QRF model's performance metrics at different threshold levels. The table also presents the average time required to predict a hypoglycemic event at different threshold levels.

**Table 5:** Performance of the quantile regression forest model at different thresholds and the average time to predict a hypoglycemic event.

Metric	30-minute prediction horizon				60-minute prediction horizon		
	Threshold 1	Threshold 2	Threshold 3	Threshold 1		Threshold 2	Threshold 3
Sensitivity (%)	98.54	99.27	99.51	97.72		98.29	98.99
Specificity (%)	98.57	97.56	96.68	98.49		97.06	95.53
False alerts (n)	6932	11,960	16,049	7215		14,027	21,297
False alerts with transient events as positives (n)	3736	8149	12,007	3974		9956	16,775
False alert rate (%)	22.36	35.36	43.34	22.44		37.19	46.96
Average time to predict an event (minutes)	18.78	22.95	26.51	25.24		35.08	48.35

## Discussion

### Principal Findings

We present a robust prediction model for providing high-quality alerts for sustained hypoglycemic risk in patients with type 1 diabetes. The final model (QRF model) was demonstrated to be robust to different validation approaches that best represent real-world application scenarios (new patients and new time periods). The primary research contributions of this work are (1) the development of a prediction model that focused on sustained hypoglycemic events and resulted in high sensitivity, high specificity, and a low FAR; and (2) improved generalizability of the model to new patients and new time periods. The model makes use of only CGM data in the past 4 hours and contextual information about the current hour of the day and day of the week to make predictions. A methodology contribution is the use of glucose predictions at multiple time points to facilitate inference of sustained hypoglycemia. The model was built using data collected from 110 patients over a range of 30 to 90 days under normal living conditions, ensuring validity of the results. The QRF model proposed in this work had sensitivity and specificity >97% for both 30- and 60-minute PHs. The FAR was also kept low at 22% and 29% for 30-minute and 60-minute PHs, respectively, which will lead to improved user trust in and adoption of CGM-based alerts.

### Comparison with the Literature

A comparative analysis of different hypoglycemia prediction methodologies can be found in the literature [11,20,59,60]. A straightforward comparison between different hypoglycemia prediction studies is complicated due to differences in CGM sensors used, sampling intervals, and data collection (synthetically generated, controlled study, free-living conditions). In addition, different studies have used different definitions of hypoglycemia, which makes their findings difficult to compare [21,61-63]. When using a regression approach, the majority of the works present an overall root-mean-square error (RMSE) value, but accuracy pertaining to the observations in the hypoglycemic range might be more relevant. Thus, providing an overall RMSE for the entire data set could misrepresent the model's performance. On the classification side, sensitivity and specificity provide accurate information on the TPs and FPs, respectively. However, due to high-class imbalance, even a moderately high specificity can lead to a high FAR. It becomes important to consider the FAR, in addition to sensitivity and specificity, in such class-imbalanced applications.

In machine learning, a standard approach to validate prediction models is to split the data into a training set (to train the model) and a validation set (to evaluate model performance) [64]. This random partitioning of the data into training and validation subsets and repeating the process across multiple folds is called cross-validation. Studies across the literature have used different validation strategies such as random sampling [20,65-67], time-based splitting [4,5,67-69], patient-specific splitting [6,32,53,70,71], or a combination of these methods to estimate predictive model performance. Simple random sampling–based cross-validation [72,73] may not fully address the generalizability aspect of the model to new patients and new time periods. Some studies [6] using a patient-based validation strategy used a part of their test data for tuning model parameters, which affected the validity of the performance estimation. The model presented in this paper had high performance in both patient- and time-based validation methods.

Mosquera-Lopez et al [28] used a patient-specific validation approach in which patient data in the test set were exclusive from the training data. Performance was reported on metrics such as sensitivity, RMSE, and FARs. However, leveraging some of the preprocessing and postprediction error-correction steps to improve performance made it difficult to achieve similar results in a real-world setting. Also, the test performance was evaluated on a small sample size of 10 patients (in a 4-week study). This might affect the generalizability of the presented results.

Dave et al [44] recently showed good results with respect to sensitivity and specificity using a random sampling–based validation approach and a threshold of 70 mg/dL for hypoglycemia. However, it was observed that performance of this model was reduced when applied to new patients and new time periods (Table 3). In addition, even with sensitivity and specificity of >95%, the model resulted in an FAR of 80% due to a large number of nonhypoglycemic events relative to the number of hypoglycemic events. From a user experience perspective, this will lead to false alert fatigue. The model presented in this paper reduced the FAR to 22%.

Having an accurate and actionable hypoglycemia prediction model with low FARs is essential to the durability of CGM in diabetes management. Furthermore, a patient-facing hypoglycemia prediction algorithm may give patients the confidence to aim for in-range glucose values without fear of hypoglycemia, potentially leading to lower glycated hemoglobin A_1c_ (HbA_1c_) values and increased time in range. Of note, 22.3% of patients analyzed were using sensor-augmented pump therapy with a predictive low-glucose suspend feature (ie, Basal-IQ technology). Patients using this system are still at risk for hypoglycemia because of insulin on board, exercise, overdosing on carbohydrates, and/or hyperglycemia, so a notification for predicted hypoglycemia using advanced machine learning models with good performance could still be clinically useful.

### Limitations

A limitation of our approach is that transient hypoglycemic events were ignored in generating alerts. Ignoring the transient events helped the machine learning model better learn the more stable patterns of sustained events. Even though the alerts were focused on detecting sustained events, 61% of the transient events were still classified as FPs. This resulted in just 39% of the transient events (representing 13% of the total hypoglycemic events) not being detected. This trade-off was justified because transient events are not as serious as sustained hypoglycemic events. Transient events may occur because of random variations in glycemic levels (ie, noise) or temporal lag in the effect of an intervention taken by the patient (eg, consuming fast-acting carbohydrates). In either case, ignoring transient events will help in learning the stable patterns of sustained hypoglycemia. The improved FAR, sensitivity, specificity, and generalizability of the sustained hypoglycemia model presented in this paper justify this trade-off.

This study was based on patients with pediatric type 1 diabetes in the age range of 0 to 20 years using Dexcom G6 CGM devices. As such, the results are directly applicable to this population. The model may need to be recalibrated to other CGM devices such as the Guardian (Medtronic) or FreeStyle Libre (Abbott Laboratories Co.); however, the performance measures should be generalizable to other platforms provided the accuracy and frequency of incoming glucose readings remain the same. Similarly, while no specific activity profile of pediatric patients was explicitly used in the model development, the model may need to be calibrated to an adult cohort by retraining on adult CGM data [74-76]. Pediatric patients were selected as the focus of the study because of our collaboration in the United States Food and Drug Administration (FDA)–funded Southwest National Pediatric Device Innovation Consortium [77]. Additionally, there is a need for a paradigm shift in diabetes management in pediatrics to avoid risk of hypoglycemia to ameliorate parental and patient fear and move toward optimizing time in range and lowering HbA_1c_.

### Conclusions

Providing predictive alerts for hypoglycemia focused on sustained events instead of all hypoglycemic events reduces FARs and improves sensitivity and specificity. It also results in models that have better generalizability to new patients and time periods. This has important implications for sustaining CGM use and optimizing glycemic control with fewer hypoglycemic events, improved confidence, and potentially lower HbA_1c_. To that end, the predictive model presented in this paper will be implemented in a smartphone app in an upcoming clinical pilot study at Texas Children’s Hospital.

## Appendix

Multimedia Appendix 1 Patient hypoglycemia profile.

Multimedia Appendix 2 Patient pump profile.

Multimedia Appendix 3 Breakdown of sustained events by daytime and nighttime.

Multimedia Appendix 4 Breakdown of sustained and transient events.

Multimedia Appendix 5 Breakdown of sustained events.

Multimedia Appendix 6 Features extracted for prediction.

Multimedia Appendix 7 Receiver operating characteristic plot showing a comparison between different classifiers for giving out predictive alerts for 30-minute (top) and 60-minute (bottom) prediction horizons.

## Abbreviations

ARIMA: autoregressive integrated moving average
CGM: continuous glucose monitoring
FAR: false alert rate
FDA: Food and Drug Administration
FN: false negatives
FP: false positives
HbA1c: glycated hemoglobin A1c
PH: prediction horizon
QRF: quantile regression forest
RF: random forest
RMSE: root-mean-square error
ROC: receiver operating characteristic
TN: true negatives
TP: true positives
