# Supplementary material for: Improved Low-Glucose Predictive Alerts Based on Sustained Hypoglycemia: Model Development and Validation Study
Source: JMIR Diabetes. 2021 Apr 29;6(2):e26909. doi: 10.2196/26909 (PMC8120423; doi:10.2196/26909)
Supplement: Multimedia Appendix 1 [file diabetes_v6i2e26909_app1.pdf]

APPENDIX V

PATEINT HYPOGLYCEMIA PROFILE

Features	Min	Max	Median	Inter-quartile range (IQR)
Very Low (CGM < 55) (%)	0	2.50	0.17	0.38
Low (CGM < 70) (%)	0.04	10.87	1.47	2.00
Sustained episodes	1	288	30	48
Total CGM Observations	1100	24648	16669	14157
