# Supplementary material for: Improved Low-Glucose Predictive Alerts Based on Sustained Hypoglycemia: Model Development and Validation Study
Source: JMIR Diabetes. 2021 Apr 29;6(2):e26909. doi: 10.2196/26909 (PMC8120423; doi:10.2196/26909)
Supplement: Multimedia Appendix 2 [file diabetes_v6i2e26909_app2.pdf]

APPENDIX VI

PATEINT PUMP PROFILE

**Breakup of Insulin Pumps**	**Frequency**
Omnipod	64
Tandem T-Slim	4
Tandem T-Slim X2	20
Medtronic	1
Medtronic 630G	1
Medtronic 670G	1
Medtronic Minimed	1
None	18

* *The Tandem T:Slim X2 w/ Basal IQ pumps have a predictive low glucose suspend algorithm*
