# Supplementary material for: Improved Low-Glucose Predictive Alerts Based on Sustained Hypoglycemia: Model Development and Validation Study
Source: JMIR Diabetes. 2021 Apr 29;6(2):e26909. doi: 10.2196/26909 (PMC8120423; doi:10.2196/26909)
Supplement: Multimedia Appendix 3 [file diabetes_v6i2e26909_app3.pdf]

Appendix

APPENDIX I
BREAKUP OF SUSTAINED EVENTS BY DAYTIME AND NIGHTTIME

5 minutes	10 minutes	15 minutes	20 minutes	25 minutes	30 minutes	35 minutes	40 minutes	45 minutes	>45 minutes	Total	Time
403 (9%)	585 (13%)	700 (16%)	649 (15%)	508 (12%)	450 (10%)	296 (7%)	203 (5%)	146 (3%)	455 (10%)	4395	Day
169 (10%)	211 (13%)	185 (11%)	193 (12%)	168 (10%)	112 (7%)	95 (6%)	80 (5%)	63 (4%)	339 (21%)	1615	Night

APPENDIX II

BREAKDOWN OF TRANSIENT AND SUSTAINED EVENTS

Event type	Rate of Event (per day)	Total
Transient	0.15	1368
Sustained	0.53	4642

APPENDINX III

BREAKDOWN OF SUSTAINED EVENTS

Time	Rate of Event (per day)	Total Events
Total	0.53	4642
Day	0.39	3407
Night	0.14	1235

APPENDIX IV

FEATURES EXTRACTED FOR PREDICTION

Variable	Description
**SHORT TERM FEATURES**	
glucose	Actual CGM observation made at a point
diff_10	Difference between current CGM observation and the one observed 10 minutes earlier
**MEDIUM TERM FEATURES**	
sd_2hr	Standard deviation of CGM observations observed in the past 2 hours
sd_4hr	Standard deviation of CGM observations observed in the past 4 hours
**SNOWBALL EFFECT FEATURES**	
pos	Sum of all increments in adjacent CGM observations in last 2 hours
max_pos	Maximum increase in adjacent CGM observations in past 2 hours
max_neg	Maximum decrease in adjacent CGM observations in past 2 hours
**CONTEXTUAL FEATURES**	
hour	Hour of the day when observation was made
day	Day of the week when observation was made

APPENDIX V

PATEINT HYPOGLYCEMIA PROFILE

Features	Min	Max	Median	Inter-quartile range (IQR)
Very Low (CGM < 55) (%)	0	2.50	0.17	0.38
Low (CGM < 70) (%)	0.04	10.87	1.47	2.00
Sustained episodes	1	288	30	48
Total CGM Observations	1100	24648	16669	14157

APPENDIX VI

PATEINT PUMP PROFILE

**Breakup of Insulin Pumps**	**Frequency**
Omnipod	64
Tandem T-Slim	4
Tandem T-Slim X2	20
Medtronic	1
Medtronic 630G	1
Medtronic 670G	1
Medtronic Minimed	1
None	18

* *The Tandem T:Slim X2 w/ Basal IQ pumps have a predictive low glucose suspend algorithm*

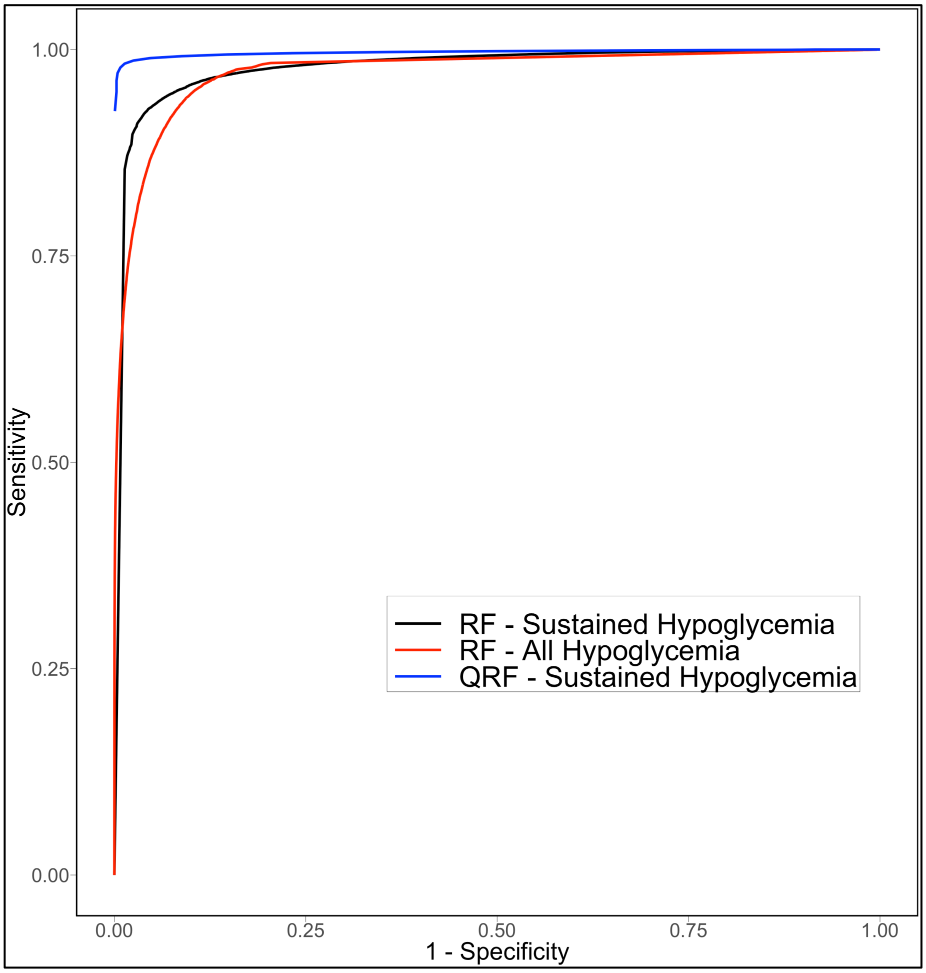

1.00
0.75
0.50
0.25
0.00
Sensitivity
0.00
0.25
0.50
0.75
1.00
1 - Specificity
RF - Sustained Hypoglycemia
RF - All Hypoglycemia
QRF - Sustained Hypoglycemia

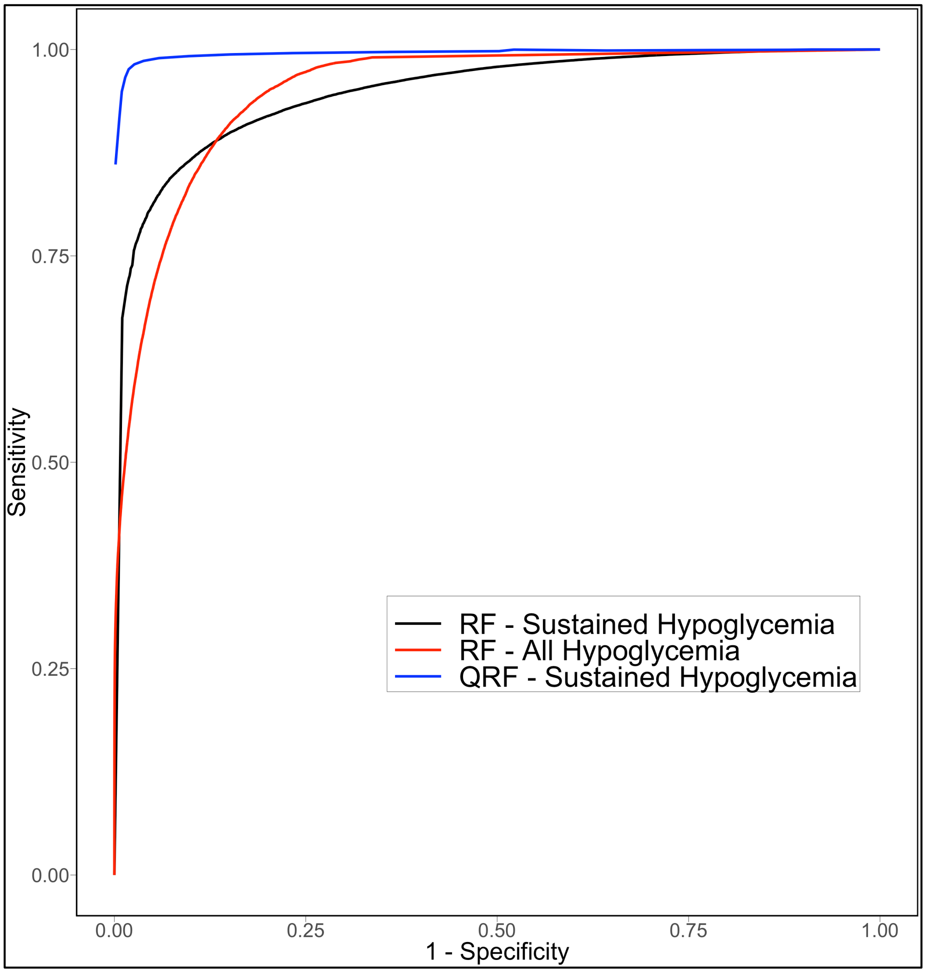

Appendix VII: ROC plot showing a comparison between different classifiers for giving out predictive alerts for (Top)30-minutes and (Bottom) 60-minutes prediction horizon
