# Supplementary material for: Improved Low-Glucose Predictive Alerts Based on Sustained Hypoglycemia: Model Development and Validation Study
Source: JMIR Diabetes. 2021 Apr 29;6(2):e26909. doi: 10.2196/26909 (PMC8120423; doi:10.2196/26909)
Supplement: Multimedia Appendix 4 [file diabetes_v6i2e26909_app4.pdf]

APPENDIX II

BREAKDOWN OF TRANSIENT AND SUSTAINED EVENTS

Event type	Rate of Event (per day)	Total
Transient	0.15	1368
Sustained	0.53	4642
