# Supplementary material for: Improved Low-Glucose Predictive Alerts Based on Sustained Hypoglycemia: Model Development and Validation Study
Source: JMIR Diabetes. 2021 Apr 29;6(2):e26909. doi: 10.2196/26909 (PMC8120423; doi:10.2196/26909)
Supplement: Multimedia Appendix 5 [file diabetes_v6i2e26909_app5.pdf]

APPENDINX III

BREAKDOWN OF SUSTAINED EVENTS

Time	Rate of Event (per day)	Total Events
Total	0.53	4642
Day	0.39	3407
Night	0.14	1235
