# Supplementary material for: Improved Low-Glucose Predictive Alerts Based on Sustained Hypoglycemia: Model Development and Validation Study
Source: JMIR Diabetes. 2021 Apr 29;6(2):e26909. doi: 10.2196/26909 (PMC8120423; doi:10.2196/26909)
Supplement: Multimedia Appendix 6 [file diabetes_v6i2e26909_app6.pdf]

APPENDIX IV

FEATURES EXTRACTED FOR PREDICTION

Variable	Description
**SHORT TERM FEATURES**	
glucose	Actual CGM observation made at a point
diff_10	Difference between current CGM observation and the one observed 10 minutes earlier
**MEDIUM TERM FEATURES**	
sd_2hr	Standard deviation of CGM observations observed in the past 2 hours
sd_4hr	Standard deviation of CGM observations observed in the past 4 hours
**SNOWBALL EFFECT FEATURES**	
pos	Sum of all increments in adjacent CGM observations in last 2 hours
max_pos	Maximum increase in adjacent CGM observations in past 2 hours
max_neg	Maximum decrease in adjacent CGM observations in past 2 hours
**CONTEXTUAL FEATURES**	
hour	Hour of the day when observation was made
day	Day of the week when observation was made
