# Supplementary material for: Improved Low-Glucose Predictive Alerts Based on Sustained Hypoglycemia: Model Development and Validation Study
Source: JMIR Diabetes. 2021 Apr 29;6(2):e26909. doi: 10.2196/26909 (PMC8120423; doi:10.2196/26909)
Supplement: Multimedia Appendix 7 [file diabetes_v6i2e26909_app7.pdf]

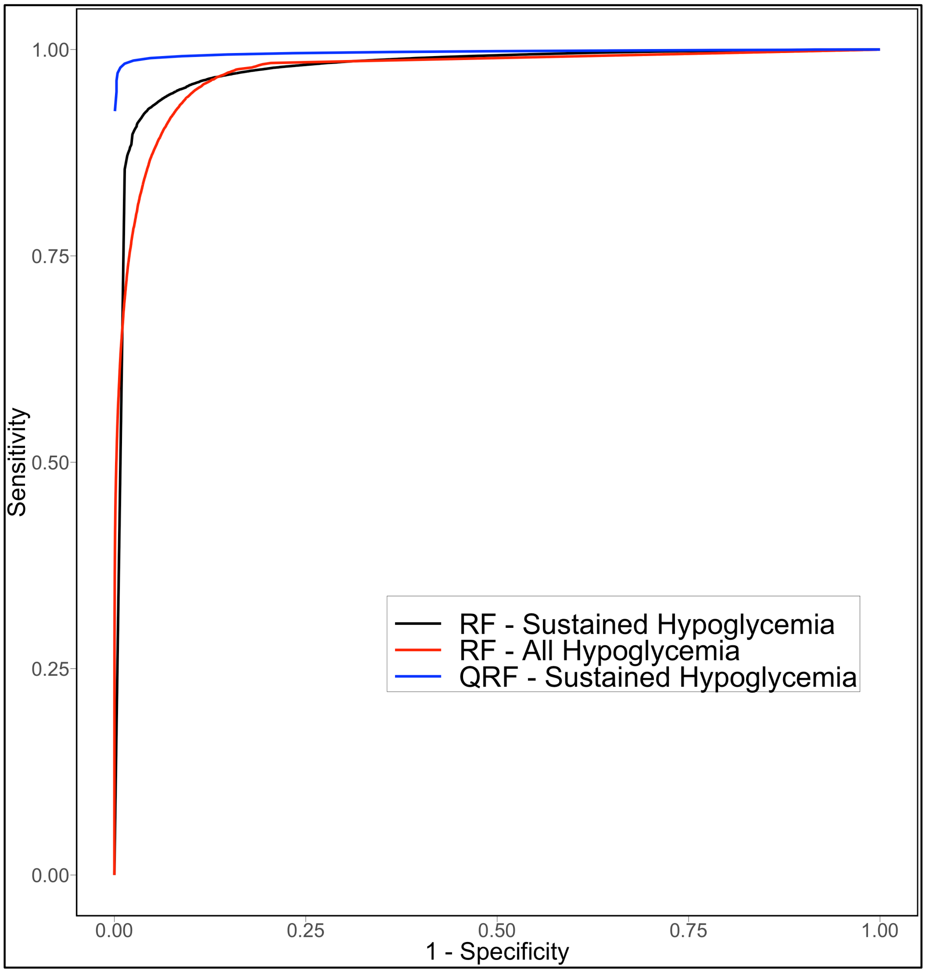
Sensitivity
1.00
0.75
0.50
0.25
0.00
0.00
0.25
0.50
0.75
1.00
1 - Specificity
RF - Sustained Hypoglycemia
RF - All Hypoglycemia
QRF - Sustained Hypoglycemia

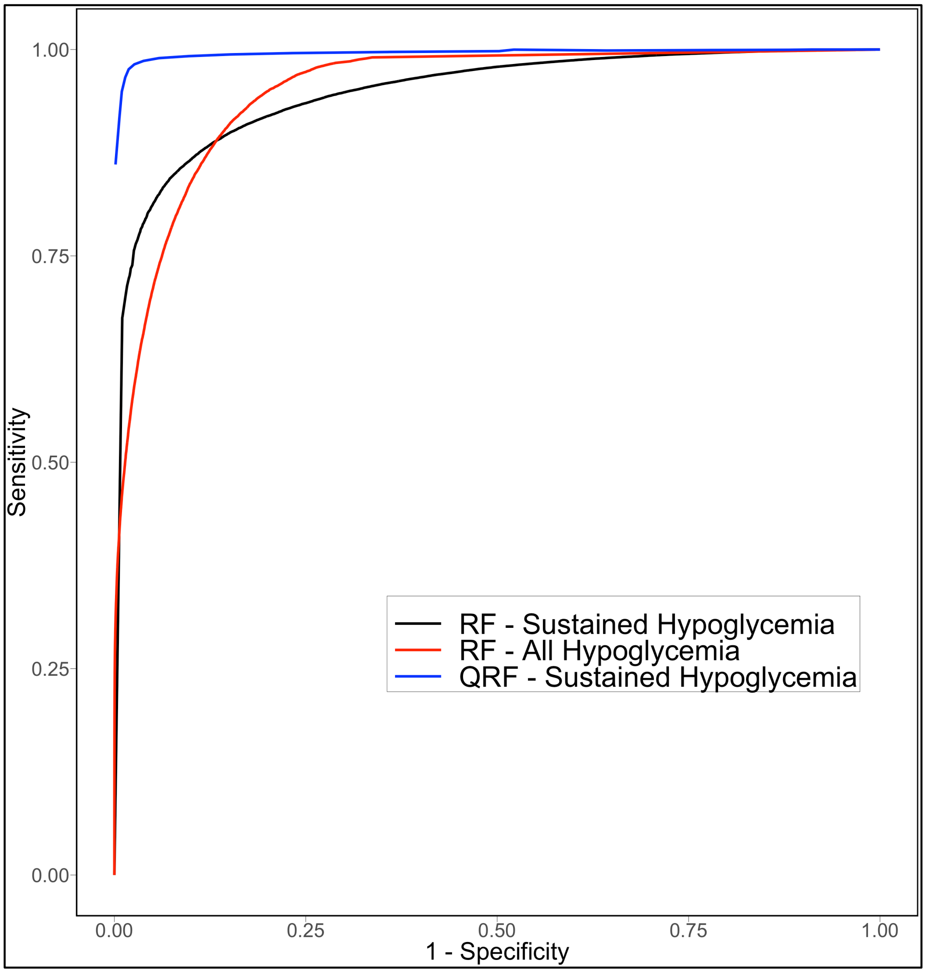

Appendix VII: ROC plot showing a comparison between different classifiers for giving out predictive alerts for (Top)30-minutes and (Bottom) 60-minutes prediction horizon
